# Supplementary material for: Preparation of Multicolor Photoluminescent Carbon Dots by Tuning Surface States
Source: Nanomaterials (Basel). 2019 Apr 3;9(4):529. doi: 10.3390/nano9040529 (PMC6523770; doi:10.3390/nano9040529)
Supplement: Supplementary file 1 [file nanomaterials-09-00529-s001.pdf]

Supplementary Materials

Preparation of Multicolor Photoluminescent Carbon Dots by Tuning Surface States

Kai Jiang [1,2], Xiangyu Feng [2], Xiaolu Gao [2], Yuhui Wang [2], Congzhong Cai [1], Zhongjun Li [3] and Hengwei Lin [2,*]

[1] State Key Laboratory of Coal Mine Disaster Dynamics and Control, Department of Applied Physics, Chongqing University, Chongqing 400044, China; jiangkai@nimte.ac.cn (K.J.); czcai@cqu.edu.cn (C.C.)

[2] Key Laboratory of Graphene Technologies and Applications of Zhejiang Province & Ningbo Institute of Materials Technology & Engineering (NIMTE), Chinese Academy of Sciences (CAS), Ningbo 315201, China; fengxiangyu@nimte.ac.cn (X.F.); gaoxiaolu@nimte.ac.cn (X.G.); wangyuhui@nimte.ac.cn (Y.W.)

[3] College of Chemistry and Molecular Engineering, Zhengzhou University, Zhengzhou 450001 China; lizhongjun@zzu.edu.cn

* Correspondence: linhengwei@nimte.ac.cn; Tel.: +86-574-8668-5130

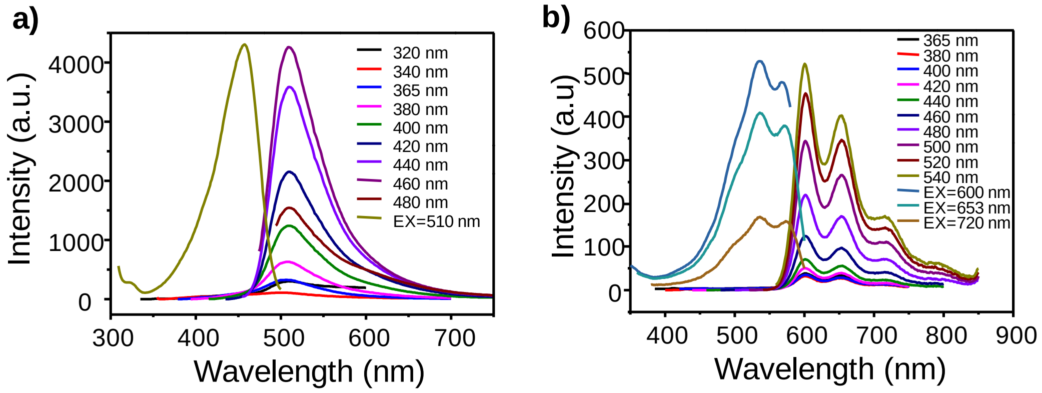

Figure S1 a) PL emission spectra of the g-CDs ethanol dispersion under different excitation wavelengths and PL excitation spectrum at emission wavelength of 510 nm; b) PL emission spectra of the r-CDs ethanol dispersion under different excitation wavelengths and PL excitation spectra at emission wavelengths of 600, 653 and 720 nm.

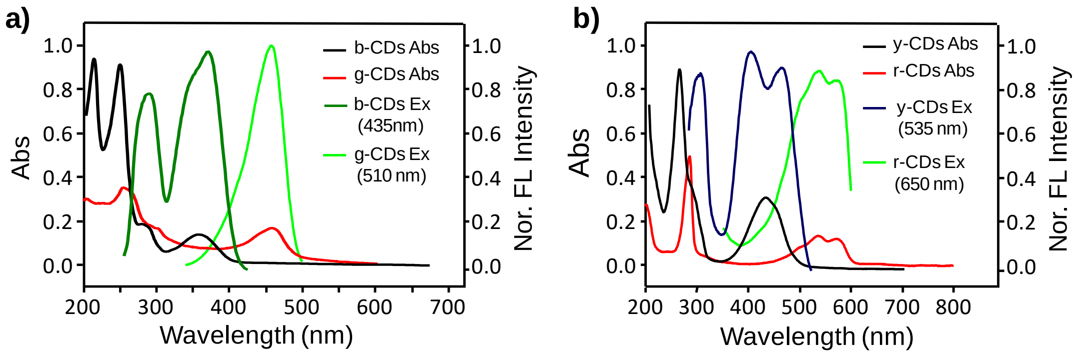

Figure S2 a) UV-Vis absorption and PL excitation spectra of b-CDs and g-CDs (excitation wavelengths at 435 and 510 nm, respectively); b) UV-Vis absorption and PL excitation spectra of y-CDs and r-CDs (excitation wavelengths at 535 and 650 nm, respectively).

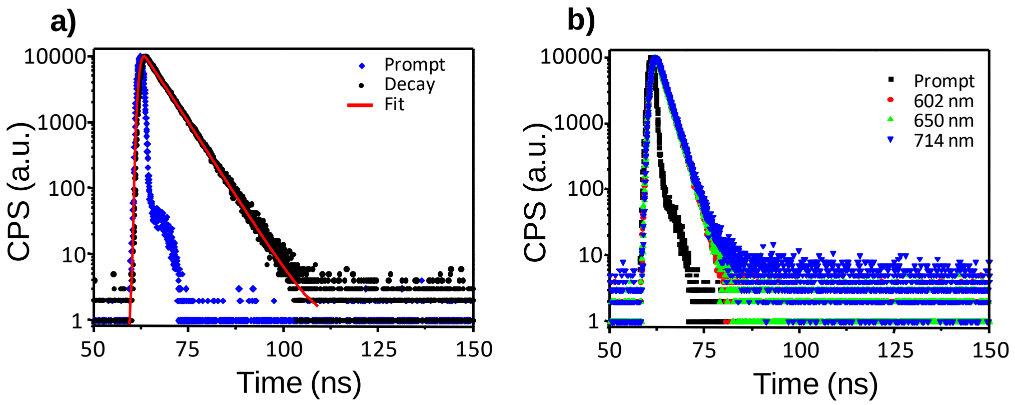

Figure S3 a) PL decay spectra of the g-CDs ethanol dispersion monitored at 510 nm under excitation of 457 nm; b) PL decay spectra of the r-CDs ethanol dispersion monitored at 602, 650 and 714 nm under excitation of 588 nm.

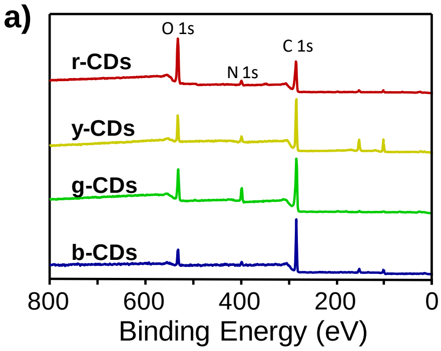

b)

Sample	C (%)	N (%)	O (%)	O/C
b-CDs	84.56	3.69	11.76	0.14
g-CDs	69.68	11.69	18.63	0.27
y-CDs	76.74	7.32	15.94	0.21
r-CDs	67.25	5.37	27.39	0.41

Figure S4 a) XPS survey of the b-CDs, g-CDs, y-CDs, and r-CDs; b) Relative contents of C, N, and O elements of these CDs based XPS data.

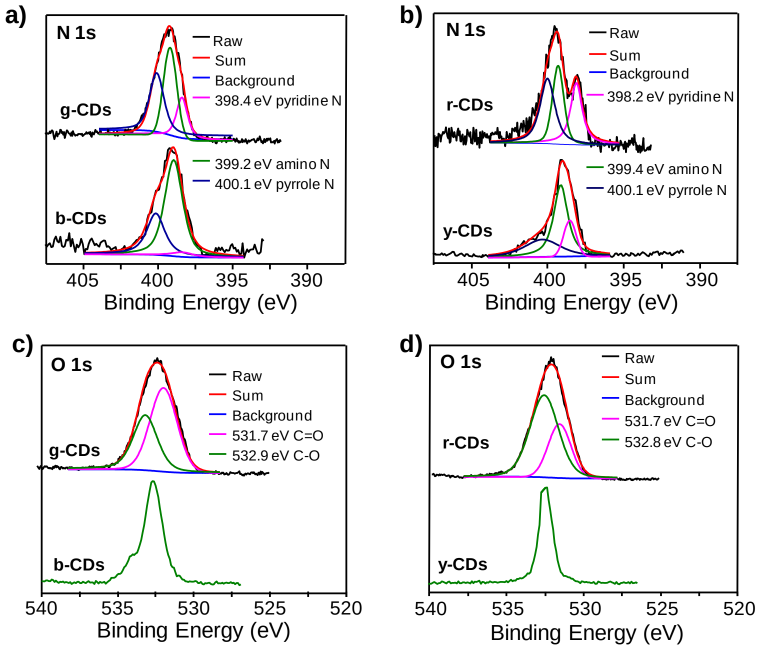

Figure S5 a-b) high resolution XPS N 1s spectra of g-CDs and r-CDs and their comparison to b-CDs and y-CDs, respectively; c-d) high resolution XPS O 1s spectra of g-CDs and r-CDs and their comparison to b-CDs and y-CDs, respectively.

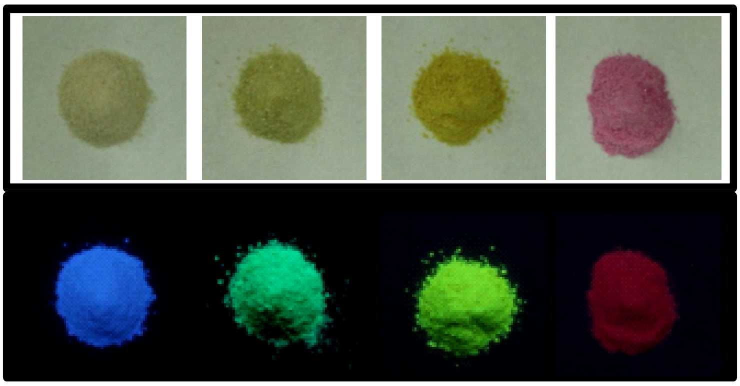

Figure S6 Photographs of the b-CDs@PVP, g-CDs@PVP, y-CDs@PVP, and r-CDs@PVP powders (from left to right) under daylight and UV light (365 nm).

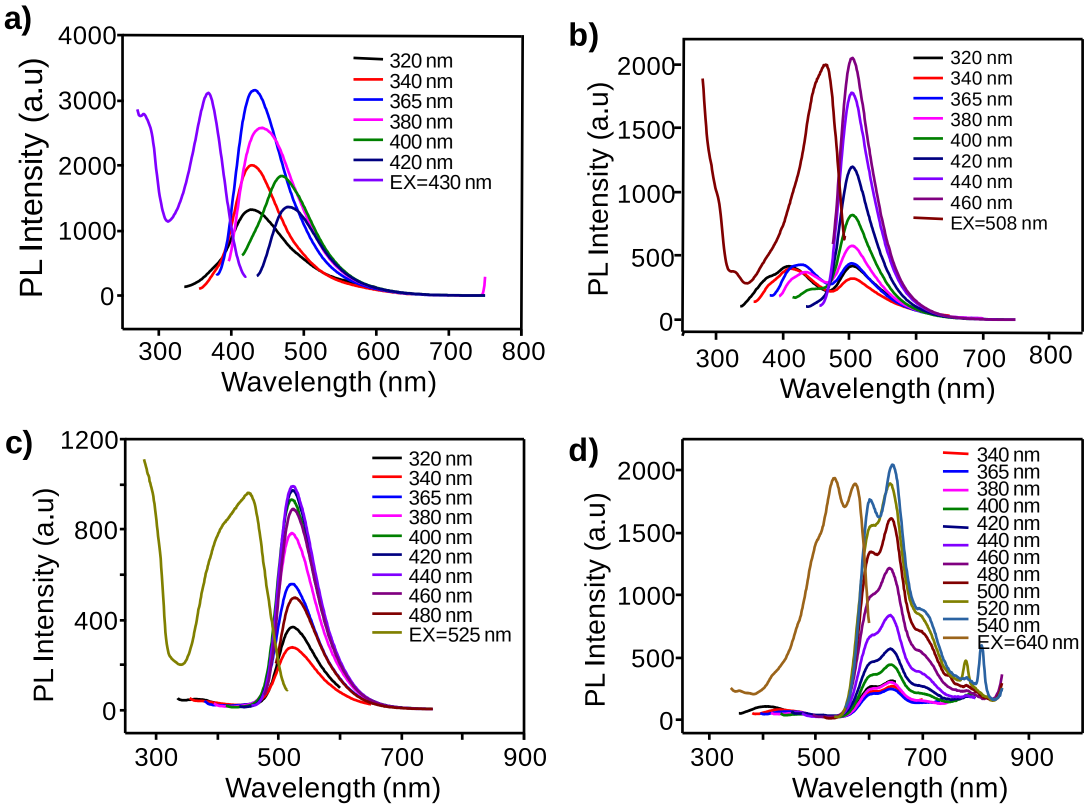

Figure S7 PL emission spectra under different wavelengths and excitation spectra at the corresponding emission maxima of b-CDs@PVP (a), g-CDs@PVP (b), y-CDs@PVP (c), and r-CDs@PVP (d) powders.

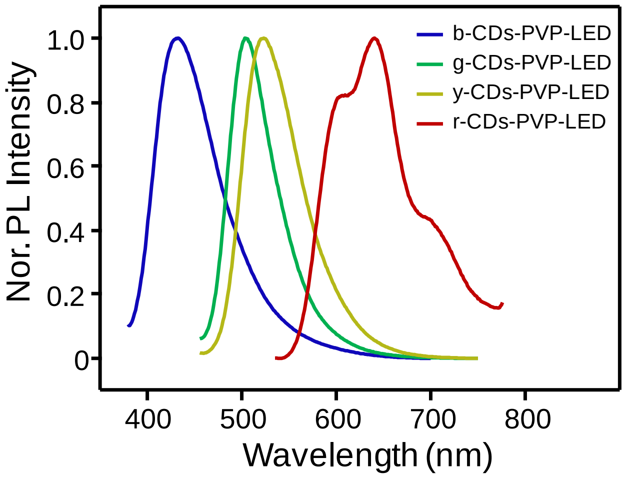

Figure S8 Corresponding emission spectra of the multicolour LEDs.

Table S1. QYs of the g-CDs and r-CDs in ethanol.

Sample	λ_{ex}(nm)	Φ_1(%)	Φ_2(%)	Φ_3(%)	Φ_4(%)	Φ_5(%)	Φ_{avg}(%)	Φ_{corr}(%)
**R-6G**	488	93.45	94.33	93.18	94.02	94.27	93.85	**95**
**g-CDs**	440	28.21	28.02	27.91	27.75	27.50	27.88	**28.22**
**r-CDs**	540	21.09	23.74	21.47	21.14	21.30	21.75	**22.01**

Table S2. Fitted parameters of the PL decay spectra of the g-CDs and r-CDs.

Sample	λ_{ex}(nm)	λ_{em}(nm)	τ_1(ns)	B_1(%)	ϕ
**g-CDs**	457	510	4.86	100	1.067
**r-CDs**	588	608	2.18	100	1.096
**r-CDs**	588	650	2.20	100	1.133
**r-CDs**	588	714	2.24	100	1.057

Table S3. PL QYs of the b-CDs@PVP, g-CDs@PVP, y-CDs@PVP and r-CDs@PVP powders.

Sample	λ_{ex} (nm)	Φ_1	Φ_2	Φ_3	Φ_4	Φ_5	Φ_{avg}
**b-CDs-PVP**	360	9.42	8.86	9.01	8.04	8.77	**8.82**
**g-CDs-PVP**	450	15.36	14.93	14.74	14.03	13.50	**14.44**
**y-CDs-PVP**	430	16.19	16.06	16.25	15.97	16.18	**16.13**
**r-CDs-PVP**	540	15.74	14.94	14.78	14.39	14.20	**14.81**
